# Genetic assessment of the variation and distribution of the species of *Salinator* (Panpulmonata: Amphibolidae) in south-eastern Australia

**DOI:** 10.3897/BDJ.8.e54724

**Published:** 2020-10-29

**Authors:** Donald Colgan, Hugo Lumsdaine

**Affiliations:** 1 The Australian Museum, Sydney, Australia The Australian Museum Sydney Australia

**Keywords:** 28S ribosomal RNA, cytochrome *c* oxidase subunit I, *Salinator fragilis*, *Salinator rhamphidia*, *Salinator tecta*, Tasmania

## Abstract

Amphibolidae is one of the most abundant families of gastropods in estuarine environments of south-eastern Australia. However, the range limits of the species of *Salinator*, one of the family’s two genera in the region, remain unclear partly due to uncertainty of identifications based solely on shell morphology. Insufficient data have been collected to address questions regarding the genetic variability of any of the species of *Salinator*. Here, DNA sequences from a segment of the cytochrome *c* oxidase subunit I and 28S ribosomal RNA genes were collected to investigate the distribution and variation of the three *Salinator* species in the region, these being *S. fragilis*, *S. rhamphidia* and *S. tecta*. The results demonstrate a large range extension in *S. rhamphidia* and suggest that *S. tecta* may have limited distribution in Tasmania. In contrast to previously-studied estuarine Mollusca in the south-eastern coasts of the mainland and Tasmania, *S. rhamphidia* has regional differentiation. There is evidence of genetic disequilibrium within *S. fragilis*, suggesting that it may presently comprise contributions from two distinct sets of populations.

## Introduction

The two genera of mudflat snails of the family Amphibolidae Gray, 1840 in southern and eastern Australia include some of the most abundant molluscs of estuarine environments in the region (Golding et al. 2007). These two genera, *Salinator* Hedley, 1900 and *Phallomedusa* Golding, Ponder & Byrne, 2007, are apparently endemic, although the former has previously been used to include a number of species from other countries that do not actually belong to it (Golding 2012). Phylogeographic studies of *Phallomedusa* (Golding et al. 2011) have confirmed the genetic differentiation of its two species and revealed the presence of two deeply-divergent intra-specific clades within *P. solida* that are both found in most of the species’ range from Tasmania to northern New South Wales. There has been no comparable study of genetic variation in any of the three *Salinator* species, *S. fragilis* (Lamarck, 1822), *S. rhamphidia* Golding, Ponder & Byrne, 2007 and *S. tecta* Golding, Ponder & Byrne, 2007, occurring in the region. Published molecular data are limited to only a few specimens of each species although, notably, the complete mitochondrial DNA of *S. rhamphidia* has been sequenced (White et al. 2011).

The morphologies of *S. fragilis*, *S. rhamphidia* and *S. tecta* are similar and the shell characters potentially distinguishing them are limited to the height of the spire and shell size (Golding et al. 2007). *Salinator rhamphidia* is smaller than the other two species. Golding et al. (2007) did not remark on any shell characters diagnostic for *S. fragilis* and *S. tecta*, which are primarily distinguished by pigmentation patterns on the animals’ heads and the structure of the spermovipositor. Golding et al. (2007) found *S. rhamphidia* only in a restricted range near Sydney, surmising that it has a wider distribution. Subsequent collection has suggested that the species occurs in Queensland (Golding, unpublished Australian Museum Malacology collection identification). Golding has also identified a lot (AMS C.445407) comprising four dead, worn shells collected in 1975 from Pambula Lake in southern New South Wales as belonging to this species. No genetic identification has been made of any specimen outside the Sydney region, although a sequence from a specimen of an unidentified *Salinator* species collected in southern Queensland (Dinapoli et al. 2011) apparently belongs to this taxon (see below).

This study was conducted to clarify the distribution and variation of the three south-eastern Australian *Salinator* species, *S. fragilis*, *S. rhamphidia* and *S. tecta*, using genetic data. The study utilised newly-collected sequences from the mitochondrial cytochrome *c* oxidase subunit I (COI) and the nuclear 28S ribosomal RNA (28S rRNA) genes and available COI sequences from GenBank.

## Materials and Methods

### Materials

Specimens were collected by hand and stored at -80°C or in 95% ethanol until use. Specimens were morphologically identified using a stereomicroscope according to the body pigmentation criteria specified in Golding et al. (2007).

**Abbreviations**

AMS: Australian Museum Malacology collection.

MAL#: designation of Australian Museum Malacology collection locality

CASIZ: California Academy of Sciences Invertebrate Zoology collection

**New collection localities and population designations**

See Fig. 1

New South Wales:

N1: Port Jackson, Exile Bay, Bayview Park (MAL# 88194), 33°50'37'' S, 151°6'54'' E, 8 Sept 2005.

N2: Cuttagee Lake, estuary to west of the road bridge (MAL#88542), 36°29'16" S, 150°03'04" E, 19/08/2009.

Pittwater, Careel Bay, (MAL# 88529), 33°37'21'' S, 151°19'56'' E, 8/03/2007 (outgroup only)

Tasmania:

T1: Tamar River, west bank, mudflats at Deviot (MAL# 72693), 41°15' S, 146°56' E, 4 April 2007.

T2: Henderson Lagoon, south bank at Falmouth (MAL# 76559), 41°30' S, 148°16' E, 3 April 2007

T3: Snug, mudflats, estuary immediately S of town, upstream of bridge (MAL# 76557), 43°4'23'' S, 147°15'22'' E, 2 April 2007.

**GenBank Accessions**

*S. tecta*: JF439218 (Golding et al. 2011) Victoria, Aireys Inlet, 38°27'54" S, 144°5'38" E

*S. fragilis*: JQ228488: C.472898 (Golding 2012) (see details above for MAL# 76559); JQ228489: C.472900 (Golding 2012), from MAL# 72693 (details above).

*S. rhamphidia*: JN620539 (White et al. 2011), NSW, Church Point, 33°39'10"S, 151°17'12"E (CASIZ 180470, lot of 18 specimens); HQ660003 (Dayrat et al. 2011), NSW Church Point 33°39'10"S, 151°17'12"E; JX680976 (Colgan and Da Costa 2013a), (MAL# 72693); GU331961, (Dinapoli et al. 2011), Australia, Queensland, Dunwich; JQ228487 (Golding 2012) Australia, New South Wales, Careel Bay, Pittwater, 33°37' S, 151°19' E (AMS C.472894).

*S. rosacea*: JQ228476 (AMS C.463440) and JQ228475 (AMS C.472902) both from Golding (2012), Australia, Northern Territory; EF489381 (Klussmann-Kolb et al. 2008), Australia, Northern Territory.

### Methods

DNA was extracted using the QIAGEN DNeasy Blood and Tissue Extraction kit following the manufacturer’s instructions. PCR amplifications generally followed the procedures in Golding et al. (2011) except that the QIAGEN TopTaq kit was used. Reactions were performed in 50 µl of a solution comprising 25 µl Top Taq master mix (with 1.25 units of TopTaq DNA polymerase), 18.75 µl water, 5 µl 10x concentrate of CoralLoad, 0.125 µl of each primer (at 100 pM/µl) and 1 µl DNA.

Data were collected from parts of the COI and 28S rRNA genes. COI was amplified using the universal primers of Folmer et al. (1994) at an annealing temperature of 43–45°C. The 28S rRNA fragment was amplified using the 28SAF primer (Colgan et al. 2007) and the reverse complement of primer 28S D6 of Colgan et al. (2007) at an annealing temperature of 50°C. The reaction products were treated with ExoSAP-IT (USB Corporation, Cleveland, Ohio) in preparation for sequencing at Macrogen (Geumchun-Gu, Korea) which was conducted in both directions using the original primers singly.

Chromatograms were examined in Sequencher version 5.4.5 (Gene Codes Corporation, Ann Arbor, MI). The dataset, comprising new sequences and GenBank data, was aligned in ClustalX (Thompson et al. 1997). BioEdit (Hall 1999) was used for visual examination of data.

Various phylogenetic analyses were conducted, although only the Maximum Likelihood (ML) analyses are reported in detail below. These were performed on the CIPRES data portal (Miller et al. 2008) using the RAxML Blackbox (Stamatakis et al. 2008) with default assumptions (not using empirical data frequencies, no invariable sites). The numbers of required rapid bootstrap replicates were calculated by the majority rules extended (“MRE”) bootstopping criterion (Pattengale et al. 2010) with the cutoff threshold of 0.03 recommended by Stamatakis (2016). Trees were examined using Figtree v. 1.4.2. (http://tree.bio.ed.ac.uk/software/figtree/).

Net pairwise Kimura 2-parameter genetic distances (Kimura 1980) between groups or species were calculated in MEGA 7 (Kumar et al. 2016). The rate variation amongst sites was modelled with a gamma distribution (shape parameter = 1). All ambiguous positions were removed for each sequence pair. DnaSP ver. 5.10.01 (Librado and Rozas 2009) was used to calculate measures of genetic variability including haplotype and nucleotide diversity and Tajima's D and Fu's Fs statistics. The probability of obtaining observed values of the D statistic was reported directly by DnaSP and that of the Fs statistic was estimated by coalescent simulation in DNaSP using 1000 replicates.

Statistical parsimony analyses of the COI data were conducted with TCS 1.21 (Clement et al. 2000) using version 1.7 of the PopART interface (Leigh and Bryant 2015). Alignment positions (62) having more than five percent of sequences missing data were excluded from the analysis.

### Data Resources

The GenBank accession numbers for the newly-collected sequences are MT356194 – MT356226 for COI and MT348593 – MT348598 for 28S rRNA.

Suppl. material 1: COI_aligned_fas.fas

The alignment of the cytochrome *c* oxidase subunit I sequences used here. Sequences are identified by the Australian Museum registration number or GenBank accession number.

Suppl. material 2: 28SA_aligned_names.fas

The alignment of the 28S ribosomal sequences used here. Sequences are identified by the Australian Museum registration number or GenBank accession number.

## Results

**Figure 2.**
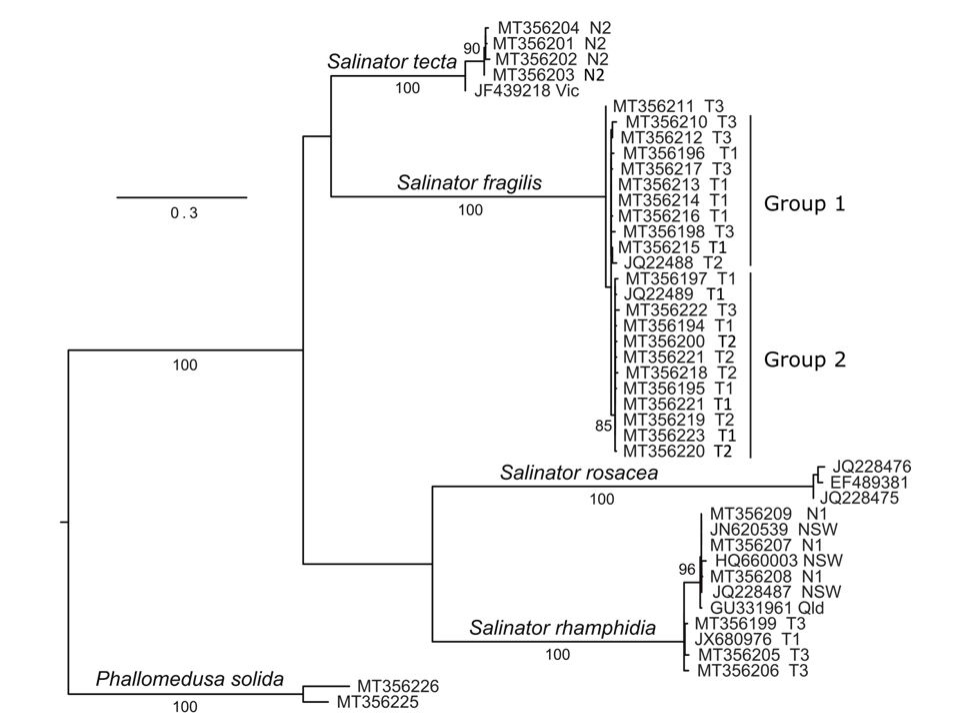
Maximum Likelihood phylogeny of the relationships between *Salinator* species from the RAxML analysis. Bootstrap percentages above 70% are shown above or below branches. Lines to the right of the sequence names specify groups 1 and 2, the two major sub-networks recovered amongst *S. fragilis* in the TCS analysis of the data. Sequences are identified by the GenBank accession number followed by a geographic locator. Sequences collected here are identified by N1 (see Materials and Methods for details) for individuals from Exile Bay, N2 for those from Cuttagee Lake, T1 from Deviot, T2 from Falmouth and T3 from Snug. Sequences from localities in other studies are located by an abbreviation of the name of the relevant State.

The alignment of COI sequences (Suppl. material 1) had a length of 655 bases, of which 481 were invariable within the group comprising *S. fragilis*, *S. rhamphidia* and *S. tecta* and 174 variable, including 164 that were parsimony informative. The RAxML analysis of these data was conducted for 708 rapid bootstraps as determined by the bootstopping criterion and the resultant topology (Fig. 2) had a *ln* Likelihood of -2531.90. Each of the three species received 100% bootstrap support in the analysis. Although *S. fragilis* and *S. tecta* were shown as sister groups, there was not strong support for any interspecific grouping within *Salinator*. Some structure was apparent within the three species. The sequences from the two populations of *S. tecta* which were sampled, were resolved as sister groups, with strong bootstrap support. The mainland samples of *S. rhamphidia* comprised a strongly-supported clade that did not include Tasmanian specimens. The latter did not form a clade in the analysis, although they are clearly more closely related to each other than to any of the mainland sequences, with a maximum Kimura 2-parameter distance of 0.011 between Tasmanian sequences compared to a minimum distance from these to mainland sequences of 0.022. The placement of the GenBank accession GU331961 (which was not assigned to a species in the database) strongly supported this specimen to belong to the species *S. rhamphidia*.

**Figure 3.**
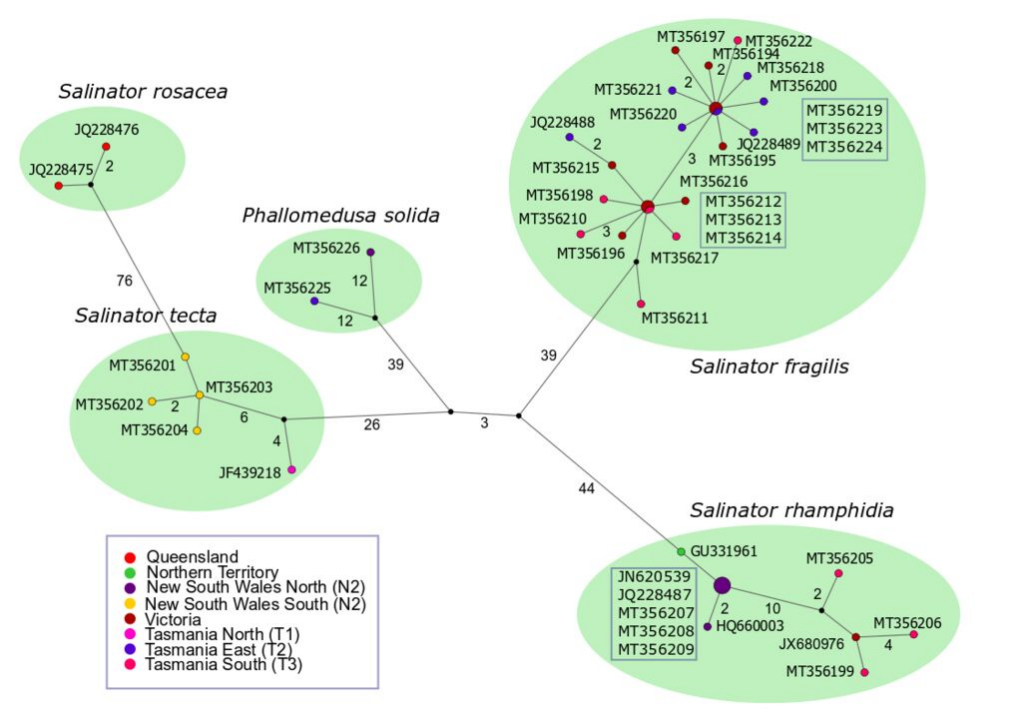
TCS analysis of the COI sequences prepared by the PopART graphical interface. The figures beside branches specify the number of mutational steps between network elements. Branches lacking figures represent one step. The provenance of sequences is colour-coded according to the legend. Abbreviations in parentheses in the legend indicate the newly-collected localities, detailed in the Materials and Methods. The numbers of occurrences of a haplotype is proportional to the size of the circle representing it. All but three haplotypes occurred only once. The specimens with a haplotype found in multiple individuals (shown by larger circles) are indicated by the accession numbers in the box nearest their symbol.

There was no geographic pattern of variation within *S. fragilis*, but there was one large clade within the species that received considerable bootstrap support (85%). TCS analysis revealed two large sub-networks in the species that were separated by three mutational changes (Fig. 3). These were designated as Group 1 and Group 2, the latter corresponding to the bootstrap-supported ML clade. Each of these two Groups had only one haplotype that was found in multiple individuals. Each of these two haplotypes were associated with multiple, closely-related haplotypes. Both Groups 1 and 2 were found in all Tasmanian populations. The sequence of AMS C.583662.001 (accession MT356211) was related to Group 1, but we have not included it in the Group as it was indirectly joined through a vertex that linked *S. fragilis* to the remainder of the network. The TCS analysis was also notable for the large number of mutational steps (minimum 11) between the mainland and Tasmanian samples of *S. rhamphidia*.

Average pairwise distances within species were similar for *S. tecta* and *S. rhamphidia*, but somewhat lower for *S. fragilis* (*Table 1*). The average distance between Tasmanian *S. rhamphidia* was 0.007 ± 0.003 which was similar to the level in *S. fragilis*. The average distance between mainland and Tasmanian samples of *S. rhamphidia* was 0.024 ± 0.006 and, within mainland specimens, it was 0.003 ± 0.001. Within *S. tecta*, the average distance between the samples from NSW and Victoria was 0.022 ± 0.006.

The genetic diversity measures (Table 2) suggest that it might be considered whether *S. fragilis* and *S. tecta* have more haplotypes and less nucleotide variability than would be expected under neutral models of molecular evolution. However, the values of Tajima’s D statistic were not significant for either species, nor was Fu’s Fs statistic significant for *S. tecta*. In contrast, this statistic did indicate significant departures from neutrality for *S. fragilis*.

The same sequence of the ribosomal RNA fragment (Suppl. material 2) was seen in both *S. tecta* (one sequence from AMS C.583693.001) and *S. rhamphidia* (two specimens from Exile Bay: AMS C.583655.001 and AMS C.583656.001 and two from Snug: AMS C.583660.001 and AMS C.583661.001). Small differences from this sequence were observed in *S. fragilis*. Two Group 2 specimens (AMS C.583675.001 and AMS C.583676.001) differed only in having a single base insert, which was heterozygous in the former and homozygous in the latter and which was not seen in any other sequences. The other four specimens of *S. fragilis* (including members of both Groups 1 and 2) differed from the *S. tecta*/*S. rhamphidia* sequence only at one polymorphic base.

## Discussion

The DNA sequencing results provide novel insights into the distribution of *Salinator* taxa in south-eastern Australia and confirm the unexpected presence of *S. rhamphidia* in Tasmania, representing a considerable extension of the species’ confirmed range. The strongly-supported inclusion of the unidentified GenBank sequence (GU331961) from Queensland in *S. rhamphidia* also represents a large extension of the genetically-confirmed range of the species. There is genetic divergence between Tasmania and mainland specimens of the species. This contrasts with previous comparisons of specimens of estuarine gastropods from Tasmania and the Australian mainland which have not identified regionally-restricted clades. The comparisons include studies of *Austrocochlea constricta* (Lamarck, 1822) (Colgan and Schreiter 2011, Colgan 2019a), *Ophicardelus ornatus* (Férussac, 1821) and *Pleuroloba quoyi* (H. Adams & A. Adams, 1855) (Colgan and Da Costa 2013b) and *Phallomedusa solida* (Martens, 1878) (Golding et al. 2011) in which, notably, both of its deeply divergent clades are found commonly in both Tasmania and the mainland’s east coast. The differences between *S. rhamphidia* specimens from Tasmania and the mainland suggest that regional partitioning of genetic variation can occur despite the homogenising effects of the East Australia Current (Murray-Jones and Ayre 1997, Colgan 2016).

Sympatry at the local level was observed for *S. tecta* and *S. rhamphidia* by Golding et al. (2007). The results here show that *S. rhamphidia* and *S. fragilis* are widely sympatric in Tasmania. The distributions of *S. fragilis* and *S. tecta* do overlap (Golding et al. 2007), but it is not confirmed that the taxa occur sympatrically. The true abundance of *S. tecta* in Tasmania cannot yet be assessed. No specimens were found amongst those sequenced here. Only one Tasmanian individual referred to as the species (AMS C.446515) has been investigated anatomically by Golding et al. (2007). Previous genetic evidence of the species presence in the State is based on a misidentification, as the sequence (JX680976) of the specimen (AMS C.467074), previously supposed to represent *S. tecta* (Colgan and Da Costa 2013a), is shown here to belong to *S. rhamphidia*.

There is considerable genetic diversity within each of the three species of *Salinator*. Statistical tests show that, at least for *S. fragilis*, this differs significantly from the expectations of neutral evolution. The negative value of the Fu’s Fs statistic for this species suggests that it has undergone recent population expansion. Such a situation has also been observed in other Mollusca in south-eastern Australia (Golding et al. 2011, Colgan and Da Costa 2013b, Colgan 2019b). One possible explanation is the isolating effect of the landbridge formation across Bass Strait at glacial maxima (Waters et al. 2005). The variation of both COI and 28S rRNA genes within *Salinator fragilis* is consistent with the suggestion that the species presently comprises genetic contributions from two or more differentiated sets of populations. This is supported by the separation of the members of the species in the TCS analysis into two star-like sub-networks. There is, however, no direct evidence as to whether the three mutational steps between the haplotypes at the centre of each "star" reflect haplotype loss during glacially-induced isolation.

## Supplementary Material

8AF5BB0A-DE77-58CA-9D4E-824027B65D17 10.3897/BDJ.8.e54724.suppl1 Supplementary material 1 COI_aligned_fas.fas Data type DNA sequence alignment Brief description The alignment of the cytochrome *c* oxidase subunit I sequences used here. Sequences are identified by Australian Museum registration number or GenBank accession number. File: oo_408505.fas https://binary.pensoft.net/file/408505 Donald Colgan and Hugo Lumsdaine

8522C760-558B-551A-97D1-C045F4DBB776 10.3897/BDJ.8.e54724.suppl2 Supplementary material 2 28SA_aligned_names.fas Data type DNA squence alignment Brief description The alignment of the 28S ribosomal sequences used here. Sequences are identified by Australian Museum registration number or GenBank accession number. File: oo_408506.fas https://binary.pensoft.net/file/408506 Donald Colgan and Hugo Lumsdaine

## Figures and Tables

**Figure 1.**
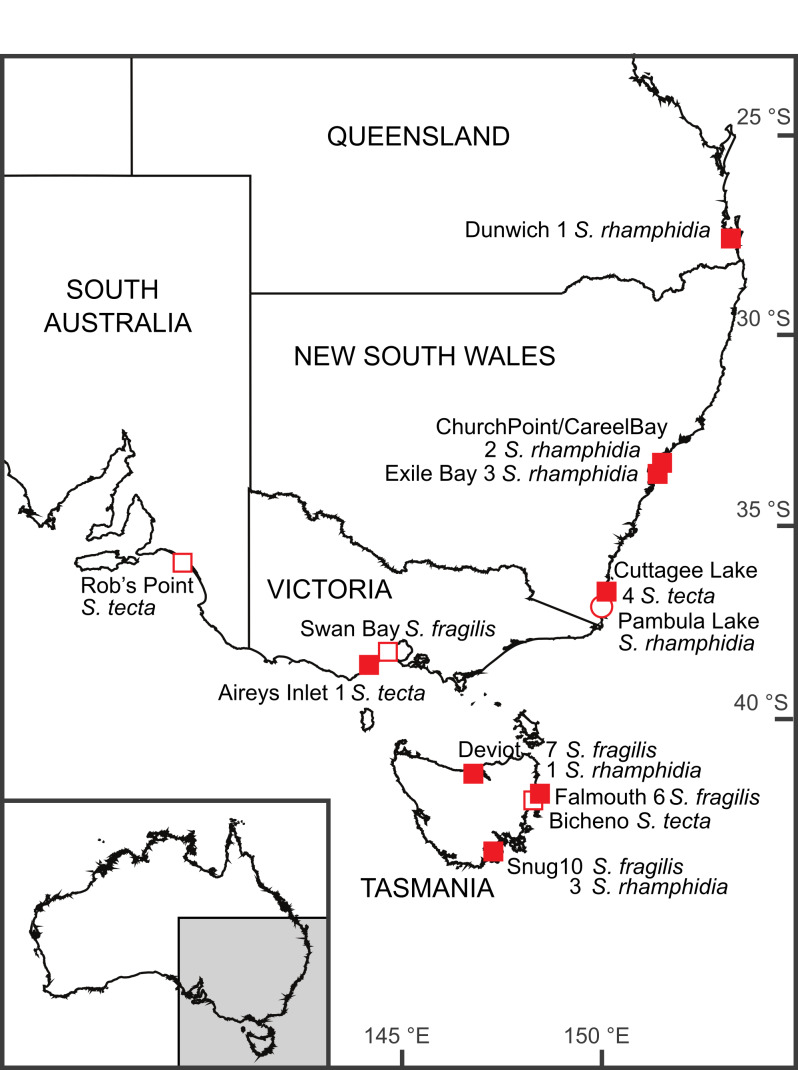
Map of collection localities of *Salinator* specimens. Details of named locations are provided in the text. Localities indicated by filled squares are represented by genetic data. Those with unfilled squares were examined using the morphology of the animal and those with open circles only conchologically. The numbers of specimens of each species’ sequences at a site are indicated next to the species name.

**Table 1.** Estimates of pairwise genetic distance within species and net pairwise genetic distance between species. Analyses were conducted using the Kimura 2-parameter model. Standard error estimates are shown above the diagonal for inter-species comparisons and after the distance measure for intra-species comparisons.

**Species**	*S. fragilis*	*S. rhamphidia*	*S. tecta*	*S. rosacea*	*P. solida*
*S. fragilis*	0.007 ± 0.002	0.244	0.021	0.028	0.027
*S. rhamphidia*	0.028	0.014 ± 0.004	0.025	0.027	0.028
*S. tecta*	0.203	0.225	0.012 ± 0.003	0.024	0.024
*S. rosacea*	0.251	0.268	0.221	0.011 ± 0.003	0.025
*P. solida*	0.224	0.243	0.206	0.212	0.055 ± 0.01

**Table 2.** Measures of genetic variability in *Salinator* species from south-eastern Australia. The columns specify the number of sequences from the species, the number of distinct haplotypes amongst these, the haplotype diversity (Hd) and the nucleotide diversity (Nd). The final two columns show the values of the Tajima’s D and Fu’s Fs statistics, with the probability that these values conform to the expectations of selective neutrality.

**Species**	n	Number of haplotypes	Hd	Nd	Tajima’s D	Fu’s Fs
*S. fragilis*	26	19	0.976	0.00757	-1.77465 0.10 > P > 0.05	-15.274 P = 0.000
*S. rhamphidia*	11	7	0.818	0.01436	0.23394 P > 0.10	0.893 P = 0.684
*S. tecta*	5	5	1.000	0.01063	-1.22485 P > 0.10	-0.875 P = 0.15246

## Hosting institution

The Australian Museum
